# Embrace open-environment machine learning for robust AI

**DOI:** 10.1093/nsr/nwad300

**Published:** 2023-11-28

**Authors:** Gang Li, Aswani Kumar Cherukuri

**Affiliations:** The Centre for Cyber Resilience and Trust, Deakin University, Australia; The Vellore Institute of Technology, India

**Keywords:** open-environment machine learning, artificial general intelligence, automated machine learning, robust AI

## Abstract

Dive into the novel OpenML paradigm, unveiling its transformative approach to robust AI in dynamic environment, shaping Automated Machine Learning with adaptability for ground breaking advancements towards Artificial General Intelligence.

Humans possess an extraordinary ability to continuously learn and apply knowledge across different environments and tasks, and this ability to understand and acquire diverse intellectual capabilities is referred to as *artificial general intelligence* (AGI). The realization of AGI requires the development of *robust artificial intelligence* systems [1] that can operate effectively and safely in complex and uncertain real-world scenarios. Despite significant advances in conventional machine learning over the last few decades, achieving robust AI or ultimate AGI remains a challenge, especially in the era where practical machine learning applications have expanded into open-environment scenarios, such as cybersecurity and multi-agent environments [2], which deviate from the assumptions of a closed environment [3]. These open scenarios pose challenges for decision-making due to various types of openness identified and the dynamic nature of the environment.

Against this background, Zhou [3] introduced *open-environment machine learning* (OpenML) as a framework to overcome the limitations of conventional machine learning approaches. While conventional approaches excel in closed environments with invariant factors—features, classes, data distributions and learning objectives—open-environment scenarios are usually confronted with varying factors, thus requiring innovative concepts, methods and techniques. Zhou emphasized the need for research on challenges such as adapting to emerging classes, changing feature space, changing data distributions and evolving learning objectives. Zhou provided clear and insightful illustrations for each dimension and offered a detailed explanation to distinguish OpenML from related efforts.

In an open-environment scenario, where each of those four dimensions—features, classes, data distributions and learning objectives—can change, machine learning tasks become very challenging due to the presence of ‘*unknown unknowns*.’ A particular challenge is dealing with new classes, where refining the trained model with available solutions may compromise its performance on known classes. To address this issue, Zhou highlighted several solutions. Another interesting aspect is the handling of incremental and decremental feature space, which requires attention in both the training and testing phases. In this regard, the One-Pass Learning with Incremental and Decremental Features (OPID) approach [4] provides a potential solution. Furthermore, dealing with varying data distributions and learning objectives is also a complex problem that requires models to adapt without new data or to simultaneously learn multiple objectives. While evolutionary mechanisms can benefit multi-objective machine learning, situations with explicit or implicit learning objectives demand new approaches. In such scenarios, the central limit theorem fails, making common sample statistics highly misleading, especially for fat-tailed distributions with rare events causing significant losses. Given the importance of minority or unknown examples, heavy-tailed distributions should be considered.

To understand the performance of machine learning algorithms in open environments, Zhou proposed a theoretical framework, which includes a robust performance measure of the form *P*(*E_i_*(*h*) ≤ ε_*i*_) ≥ 1 − ε_*i*_ for the majority region χ_1_ and the heavy-tailed region χ_2_, where *E_i_*(*h*), gε_*i*_ and σ_*i*_ are the generalization risk, the user-specified accuracy parameters and the confidence parameters in the χ_*i*_ regions, respectively. With 0 < ε_1_ ≤ ε_2_ ≤ ε, the desired model is expected to have excellent performance in region χ_1_ and satisfactory performance in region χ_2_. This is the first theoretical learning framework that enables the pursuit of high-performance learning algorithms with guaranteed results, regardless of changes that may occur in dynamic and unpredictable learning environments.

OpenML’s explicit embracing of changes across four dimensions offers a promising opportunity for *automated machine learning* (AutoML) [5]. As the data used to train AutoML systems can evolve over time, robustness and adaptability are critical requirements for AutoML systems. OpenML explicitly addresses research issues related to robustness, such as determining the conditions under which learning can be carried out in the open environment, and identifying the worst-case performance guarantees.

OpenML represents a pivotal moment in the quest for *robust artificial intelligence* and, ultimately, AGI. OpenML is transformative and offers avenues for developing new datasets, exploring novel learning techniques and creating innovative optimization models. On this transformative journey, however, researchers may need to address specific challenges, such as learning in the absence of external knowledge, effectively integrating new knowledge without compromising previously acquired knowledge and managing learning processes with multiple objectives. An in-depth exploration of these issues provides an opportunity to realize the full potential of OpenML, and advance the field of AI towards improved robustness and AGI.
